# Protective Effect of Breastfeeding on the Adverse Health Effects Induced by Air Pollution: Current Evidence and Possible Mechanisms

**DOI:** 10.3390/ijerph16214181

**Published:** 2019-10-29

**Authors:** Monika A. Zielinska, Jadwiga Hamulka

**Affiliations:** Department of Human Nutrition, Institute of Human Nutrition Sciences, Warsaw University of Life Sciences—SGGW, 159C Nowoursynowska Street, 02-776 Warsaw, Poland; jadwiga_hamulka@sggw.pl

**Keywords:** air pollutants, antioxidants, breastmilk, children, heavy metals, long chain polyunsaturated fatty acids (LC PUFA), nitrogen dioxide, ozone, particulate matter (PM), polycyclic aromatic hydrocarbons (PAHs)

## Abstract

Air pollution is a major social, economic, and health problem around the world. Children are particularly susceptible to the negative effects of air pollution due to their immaturity and excessive growth and development. The aims of this narrative review were to: (1) summarize evidence about the protective effects of breastfeeding on the adverse health effects of air pollution exposure, (2) define and describe the potential mechanisms underlying the protective effects of breastfeeding, and (3) examine the potential effects of air pollution on breastmilk composition and lactation. A literature search was conducted using electronic databases. Existing evidence suggests that breastfeeding has a protective effect on adverse outcomes of indoor and outdoor air pollution exposure in respiratory (infections, lung function, asthma symptoms) and immune (allergic, nervous and cardiovascular) systems, as well as under-five mortality in both developing and developed countries. However, some studies reported no protective effect of breastfeeding or even negative effects of breastfeeding for under-five mortality. Several possible mechanisms of the breastfeeding protective effect were proposed, including the beneficial influence of breastfeeding on immune, respiratory, and nervous systems, which are related to the immunomodulatory, anti-inflammatory, anti-oxidant, and neuroprotective properties of breastmilk. Breastmilk components responsible for its protective effect against air pollutants exposure may be long chain polyunsaturated fatty acids (LC PUFA), antioxidant vitamins, carotenoids, flavonoids, immunoglobins, and cytokines, some of which have concentrations that are diet-dependent. However, maternal exposure to air pollution is related to increased breastmilk concentrations of pollutants (e.g., Polycyclic aromatic hydrocarbons (PAHs) or heavy metals in particulate matter (PM)). Nonetheless, environmental studies have confirmed that breastmilk’s protective effects outweigh its potential health risk to the infant. Mothers should be encouraged and supported to breastfeed their infants due to its unique health benefits, as well as its limited ecological footprint, which is associated with decreased waste production and the emission of pollutants.

## 1. Introduction

Air pollution, primarily by fine particulate matter (PM_2.5_ and PM_10_, and the heavy metals therein), has been a years-long fundamental worldwide health, social, and economic issue [1,2,3]. In 2015, exposure to PM_2.5_ was already the fifth-leading mortality risk factor and contributed to 7.6% of deaths worldwide [4]. Inhabitants of low- and middle-income countries bear the highest costs of air pollution. Nearly 92% of deaths caused by air pollution occurred in these world regions [5]. Moreover, pregnant women and children are particularly vulnerable to the adverse health effects of air pollution [2,6]. Even exposure to a low dose of an air pollution during early development may cause disability, disease, or even death in early childhood or later life [5]. Unfortunately, in the last few years, air pollution has systematically increased and will keep increasing for most air pollutants, especially in South and East Asia (China and India) and less quickly in North America, Europe, and Africa, according to the Organisation for Economic Co-operation and Development (OECD) estimation [2]. As a result, by 2060, air pollution will lead to increased global economic costs (including health costs, lower labor productivity, and agriculture) at a level of 1% of global gross domestic product (GDP) [2].

A decrease in air pollution emission and human exposure to it are the main goals of environmental policies [2,4,5]. However, it is important to take action to reduce the air pollution health burden [5,7]. A review by Péter et al. [7] showed that nutrients, such as vitamins (B, C, E, D) and *n*-3 long-chain polyunsaturated fatty acids (LC PUFAs), may have protective effects against the damage induced by air pollutants. Most of the analyzed studies were conducted among adults and used dietary supplements. Unfortunately, much less is known about the potential nutritional solutions to reduce the adverse health effects of air pollution in the youngest groups. Breastfeeding is a gold standard in infant and toddler nutrition. The World Health Organization (WHO) recommends exclusive breastfeeding (EBF) for the first six months of life and further breastfeeding along with complementary feeding up to two years or more, as long as the child and mother desire [8]. Breastfeeding has well documented as having wide-ranging health benefits both for the mother and children [9], and may be able to diminish the adverse health effects of exposure to environmental pollution [10]. Thus far, several studies have investigated the effects of breastfeeding on the adverse health effects induced by air pollutants; however, the results were inconclusive [11,12,13]. 

Hence, the primary aim of this narrative review was to provide a comprehensive summary of the current evidence about the effects of breastfeeding on the adverse health effects induced by exposure to air pollutants during the first 1000 days of life. The secondary aims were an attempt to (1) define and describe potential mechanisms underlying the protective effects of breastfeeding against the influence of air pollution exposure, and (2) to examine the potential effects of air pollution on breastmilk composition and lactation.

## 2. Materials and Methods

Due to that fact that this review aims to be a comprehensive review of the evidence about interactions between breastfeeding and adverse health effects of air pollution exposure and its possible mechanisms, and is not a systematic review as per Preferred Reporting Items for Systematic Reviews and Meta-Analyses (PRISMA), in this review, we focused on the air pollutants that are the most common or related with the highest burden of diseases (e.g., PM and the heavy metals therein, ozone (O_3_), polycyclic aromatic hydrocarbons (PAHs), nitrogen dioxide (NO_x_), sulfur dioxide (SO_2_), carbon monoxide (CO), indoor air pollution).

Prior to drafting the manuscript, a search of the literature concerning the following issues covered the aims of this review, and an important background search was conducted using PubMed, ScienceDirect, Scopus, Cochrane Library, and Google Scholar with no restriction placed on publication date or country. The last search was performed in August 2019. Search strategies included various combinations of the following text words and Medical Subject Headings (MeSH) terms: air pollution (or air pollutants, air quality); environmental exposures; outdoor; indoor; particulate matter; toxic metals; polycyclic aromatic hydrocarbons; ozone; nitrogen dioxide (NO_x_); sulfur dioxide (SO_2_); carbon monoxide (CO); coal; breastfeeding; breastmilk (or breast milk, human milk); infant formula; infants; children (or childhood); mothers (or maternal); pregnancy; prenatal; lactation; postnatal; adverse effects; growth; development; psychomotor development; respiratory system; immune system; nervous system; brain; lung; allergy diseases; oxidative stress; inflammation; LC PUFA; antioxidants; vitamin A; vitamin C; vitamin E; carotenoids; flavonoids; probiotics; cytokines; immunoglobins; supplementation.

Publications were also found by scanning the references list of previous relevant studies (backward search) or by locating the most recent articles that cited the previous relevant studies (forward search). Publications published in languages other than English but with an English abstract or reported only as abstracts were eligible for inclusion only if sufficient information was available from the report. Papers that reported an analysis of the correlation between exposure to air pollutants and health outcomes were excluded from analysis when breastfeeding was only one of the adjustment variables and its influence was not described.

## 3. Findings and Discussion

### 3.1. Air Pollution Characteristics

The World Health Organization defines air pollution as the contamination of either outdoor or indoor environments by any chemical, physical, or biological agent that modifies the natural characteristics of the atmosphere [14]. Air pollution is a complex mix of both particulate and gaseous compounds [7]. Gaseous air pollutants, such as O_3_, NO_x_, CO, and SO_2_, are present as vapors or gases and are easily absorbed into the human respiratory system [14]. Meanwhile, PM air pollutants are materials in the liquid or solid phase of varying sizes suspended in the atmosphere [14]. PM consists of a variety of chemicals, including sulphates, nitrates, ammonia, sodium chloride, black carbon (BC), mineral dust, and water, but also chemicals such as PAHs (e.g., benzo(a)pyrene (B(a)P)), heavy metals (As, Cd, Pb, Ni) [1]. The main sources of outdoor air pollution are motor vehicles, industrial processes, forest fires, and biomass combustion [14,15]. Indoor air pollution, caused mostly by solid fuel combustion, tobacco smoking, poor ventilation, and household processes, is also an important health issue [14,15]. Secondary pollutants, such as O_3_ and NO_x_, may form within the atmosphere during the course of chemical reactions of pollutants emitted into the atmosphere [14].

Major constituents of outdoor air pollution are PM, O_3_, CO, NO_x_, and SO_2_, where the most problematic are PM and O_3_ [2,4,14,16]. Air quality is also dependent on the season and factors such as weather (temperature, humidity, wind) or local climate. Usually, ozone concentrations peak during the summer, whereas PM and PAHs concentrations peak during fall and winter [14,16,17]. According to the WHO data, only 16% of the assessed population is exposed to PM_2.5_ and PM_10_ annual concentrations complying with the WHO Air Quality Guide (AQG, lower than 10 or 20 µg/m^3^, respectively) [1,14]. The highest percentage of cities with PM levels exceeding the AQG levels was observed in regions of the Eastern Mediterranean, the Western Pacific, Southeast Asia (especially China and India), Africa, and European and American low- and middle-income countries [1]. The WHO stated that in the Western Pacific and Southeast Asia, about 90% of people live in an area with air that does not comply with the AQG Guidelines [1]. In the European Union, over 75% of the urban population is exposed to PM_2.5_, O_3_, and B(a)P levels above WHO AQG levels; meanwhile, exposure to PM_10_ or NO_2_ and SO_2_ is lower (around 40–50% and 10–40%, respectively) in these regions [16]. Also, North America struggles with air pollution. In the USA, around 41% of the population breathes unhealthy air [18].

### 3.2. Air Pollutant Mechanisms of Action 

The diversity of air pollution makes describing its influence on the human body and health rather difficult. Particulate matter, such as PM_10_, can reach the large upper branches but are easily caught and removed by coughing, for example, but PM_2.5_ can reach the deepest parts of the lungs and stay there if it is insoluble in water or passes into the blood if soluble [19]. Furthermore, particles smaller than 0.1 µm (ultrafine particles, UFP) are able to penetrate biological membranes, including placental and brain barriers [20,21]. In addition, UFP translocation into the brain is possible via the olfactory nerve [22]. It was also suggested that PAHs can cross the placenta [23]. Moreover, air pollutants may reach the gastrointestinal tract directly through consuming contaminated food and water or indirectly from inhaled particles with swallowed mucosa from the respiratory tract [24].

It has been shown that air pollutants are connected with a variety of negative actions in the human body that encompass mechanisms such as: (1) the induction of oxidative stress (e.g., NO_2_, O_3_, PM) [19,25,26]; (2) pro-inflammatory effects (e.g., PM, heavy metals, NO_2_) [20,25,27,28,29,30]; (3) genotoxic effects (e.g., PM, PAHs, B(a)P) [23,26,31]; (4) the induction of the coagulation system [32]; (5) the induction of epigenetic changes [19,26,33]; (6) the endocrine disruptor effect (e.g., PAHs, PM-heavy metals, NO_2_) [26,34,35]; (7) the downregulation of the amounts of antimicrobial proteins and peptides [36]; (8) the modification of the immune response [19,30,37,38]; and (9) the activation of the hypothalamus–pituitary–adrenal and sympathetic–adrenal–medullary axes [39]. It also has been suggested that air pollution may trigger a vitamin D deficiency by directly blocking ultraviolet B (UVB) photons and indirectly by decreasing outdoor activity [40]. These actions primarily affect the functioning of the immune, respiratory, and cardiovascular systems, and lead to further health effects [19,41].

### 3.3. Health Effects of Air Pollution Exposure in the First 1000 Days

The health effects of air pollutants may differ according to the source of origin and its composition [42,43]. The dosage and timing of exposure to air pollutants, which determines its harmfulness, are not without significance [41]. Also, sex, age, nutritional status, premature birth, or other predisposing conditions, such as coexisting diseases or obesity, may influence an organism’s response to the exposure and modify health outcomes [34,41,42,44,45,46,47,48,49].

Children are particularly susceptible to the negative effects of air pollution [6]. The reason behind the increased vulnerability of infants and young children to the adverse effects of air pollution is tied to several factors. First, the effect of adverse factors in the period of intensive growth and development considerably raises the risk of developmental abnormalities, especially in organs that are particularly vulnerable in this time, such as the brain or lungs [6,19,46,50,51,52]. Second, in the first years of growth, most organs and systems in the human body remain anatomically and functionally undeveloped, and in effect, the organism’s protective systems (e.g., the immune and antioxidant defense systems) are much less efficient than in adults. Third, when compared with adults, children (0–12 years) have higher metrics with respect to ventilation, absorption, and a higher ratio of body surface to body volume. Therefore, children inhale more air (and air pollution) per unit of body weight [53,54]. Fourth, children spend more time outdoors than adults. Also, due to their lower height, they are exposed to higher concentrations of some pollutants present in the air at lower heights [54]. According to the Barker hypothesis, environmental factors during fetal development and early postnatal life (the first 1000 days of life) may affect the metabolism and development and lead to increased health risks in later life [50,55]. Thus, even when exposure to air pollution does not result in clinical consequences, it is nonetheless often tied to functional disruptions and increased risk of diseases in later childhood and adulthood [19]. So far, air pollution exposure in the first 1000 days of life has been linked with a variety of adverse health effects (Table 1). 

Notwithstanding, among the range of health outcomes, adverse respiratory and neurodevelopmental outcomes, as well as an increased risk of allergic diseases, are well-documented and noticeable [6,44,82]. 

The burden of air pollution is expected to increase over the coming years, so countermeasures to decrease its health costs are necessary. As children are not only society’s future, but also its most vulnerable members, it is crucial to undertake urgent action to minimize their exposure and its negative health effects. Previously, it had been shown that breastfeeding is associated with a decreased risk of many of the aforementioned health risks and may diminish health outcomes caused by environmental pollutants [9,10]. 

### 3.4. Modifying Breastfeeding’s Effects on Health Outcomes Induced by Air Pollution

Hitherto, several studies have investigated the influence of breastfeeding on indoor air pollution health outcomes, such as respiratory tract infections, asthma, impaired mental development, and under-five mortality, mostly in low- and middle-income countries (Table 2). It has been shown that breastfed children had a decreased risk of lower respiratory infections, asthma, and under-five mortality related with exposure to indoor PM_2.5_ and air pollution from coal or cooking fuels [38,58,81]. Also, the duration of any breastfeeding was related to a lower risk of lower respiratory tract infections and better mental development despite exposure to indoor NO_2_ and air pollution from gas cooking [57,71]. The influence of current breastfeeding status on the mortality of children exposed to solids or cooking fuels was also investigated, but the results were inconclusive [79,80]. A study conducted in Nigeria [79] showed that currently breastfed children had a lower risk of neonatal and post-neonatal mortality, whereas a study conducted in 23 sub-Saharan countries [80] provided the opposite results. Owili et al. [80] explained that this may be related to a higher exposure to smoke of currently breastfed children because mothers may still carry them while cooking. 

More studies investigated the influence of breastfeeding on outdoor air pollution health outcomes, such as respiratory and neurodevelopmental outcomes of outdoor air pollution, mostly in middle-income countries (Table 3). All breastfed children had a lower risk of asthma, allergic rhinitis, and respiratory symptoms compared with never-breastfed children exposed to PM and gaseous pollutants [64]. Meanwhile, any breastfeeding for at least 6 months decreased the occurrence of asthmatic and allergic symptoms induced by PM_2.5_ [63], as well as the adverse effects of NO_2_ and benzene on mental development [72]. However, a study conducted in Switzerland showed a higher but nonsignificant negative association between PM_10_ and respiratory symptoms among breastfed infants compared with non-breastfed ones [83]. The authors explained that this may be the effect of the chemical contamination of the breastmilk of mothers exposed to air pollution. Exclusivity of breastfeeding was also studied and showed protective effects on neurodevelopment [73,74]. Mainly breastfeeding for 3 months was shown to decrease the adverse impact of air pollution on respiratory health [11], lung function [13], and blood pressure [84]. However, the duration of predominately breastfeeding showed no effect on neurodevelopmental outcomes with prenatal exposure to PM_2.5_ and NO_2_ [12]. The lack of the protective effects of breastfeeding may be explained by the fact that the effects of prenatal exposure to pollutants are higher than the protective effect of breastfeeding, or if the mother is still exposed to pollutants, she may transfer them to the infant via breastmilk [12].

The protective effects of breastfeeding on the adverse health effects induced by air pollution were confirmed in 13 out of 17 studies investigating these associations. However, only a few health results have been investigated thus far, and different breastfeeding classifications have been used. Also, it remains unclear how long the protective effects of breastfeeding persist. Several of the analyzed studies showed that the protective action of breastfeeding may diminish with age, but further studies are necessary [11,13,79]. Moreover, the exact mechanisms of modifying breastfeeding actions remain unclear; nonetheless, several possible explanations have been proposed.

### 3.5. Possible Mechanisms of Protective Breastfeeding Effects

First, it has been hypothesized that breastfeeding causes an improvement in immune function that may provide both short-term and long-term protection against infection and allergies [11,58,63,85]. Second, breastfeeding may reduce systemic inflammation [13,84]. Third, breastfeeding may reduce respiratory infection severity and morbidity, as well as postpone the age at which infections first occur, limiting lung damage. Moreover, breastfeeding may be associated with better lung functioning [13,81]. Fourth, breastfeeding has a positive influence on neurodevelopment, mainly due to breastmilk nutrients and bioactive factors, as well as its neuroprotective activity [9]. Those breastfeeding actions are related to breastmilk nutrients and bioactive factors that are multifunctional and may reduce inflammation and oxidative stress, as well as boost the immune system, such as cytokines, LC PUFA, antioxidant vitamins, and carotenoids [13,38,71,73,84]. Breastfeeding’s mitigating effect on the adverse health outcomes of other environmental factors was also reported [86].

#### 3.5.1. Breastfeeding and the Immune System

Breastfeeding appears to be protective against the development of allergic diseases, but the results are still inconclusive. Many previously conducted studies and reviews showed a negative association between breastfeeding and the occurrence of allergic diseases in infancy and later life [87]. In contrast, a strict meta-analysis conducted by Lodge et al. [85] found limited associations between breastfeeding and a reduced risk of allergic rhinitis and eczema in the first 5 or 2 years of life, respectively, and no influence on food allergies. However, the authors observed a greater protective effect of breastfeeding in low-income countries. Also, the Promotion of Breastfeeding Intervention Trial (PROBIT) did not show that breastfeeding promotion had any effect on atopic eczema at age 16 [88]. This conflicting evidence may be partly explained by variations in breastmilk composition [87].

However, a number of studies confirmed that breastfeeding is associated with lower morbidity and hospitalizations due to infectious diseases in infancy and early childhood [9,89]. Moreover, this protective effect of breastfeeding is dose-dependent and its benefits increase with its exclusivity and longer duration [9,90]. The duration and exclusivity of breastfeeding determines the total amount of immune factors received with breastmilk and may be another explanation of the mixed results. However, the concentration of many immunomodulators are the highest in colostrum; therefore, even the shortest breastfeeding duration may affect the immune system [87,91,92]. For example, Lee et al. [93] observed an improvement in immune functions after oropharyngeal colostrum administration two weeks after intervention. Previous studies suggested that breastfeeding may support both passive and active immunity during the first months and years of life [94].

The positive breastfeeding effect on the immune system is possibly due to the unique composition of breastmilk, which provides a variety of different compounds that may directly or indirectly support an infant’s ability to resist infections and promote immune system development [87,94,95,96]. Breastmilk contains compounds that improve innate immunity, e.g., lactoferrin, lysozyme, oligosaccharides, microbiome, or cluster of differentiation 14 (CD14), as well as cellular components [87,94,96,97]. Active immunity is boosted through breastmilk immunoglobins A, M, and G (IgA, IgM, IgG, respectively). The main breastmilk immunoglobulin, secretory immunoglobin A (sIgA), is crucial for passive antimicrobial protection, especially against enteric and respiratory pathogens [94]. A study conducted by Orivuori et al. [91] suggested that higher levels of sIgA may be protective against eczema in the first 2 years of life, but this effect diminished up to the age of 6 years. Also, breastmilk contains many immunomodulatory substances, including cytokines or nutrients such as LC PUFA; vitamins A, D, B_12_; and zinc [87,94]. Transforming growth factor β (TGF-β) promotes IgA production, regulates inflammation, and promotes oral toleration [98]. However, a recent systematic review did not find strong associations between breastmilk tumor necrosis factor β (TNF-β) and allergic outcomes in infancy and childhood [92].

It was shown that *n*-3 LC PUFAs have the ability to stabilize T-cell membranes and improve T-cell signaling, as well as reduce the production of pro-inflammatory substances, whereas *n*-6 LC PUFAs enhance its production [99,100]. Despite that, the associations between breastmilk LC PUFAs and allergic disease outcomes were not confirmed by a systematic review [101]. Moreover, it was shown that maternal fish intake during pregnancy diminished the adverse effects of exposure to PM_2.5_ in the second trimester of the gestation period by reducing the risk of symptoms of infantile eczema, wheeze, and cough [102,103]. Infant supplementation with fish oil may also protect from pro-allergic sensitization induced by traffic-related air pollution [104]. It was also shown that *n*-3 LC PUFA intake in asthmatic children reduced the effect of indoor PM_2.5_, whereas *n*-6 LC PUFA intake was associated with an amplified effect of indoor PM_2.5_ on symptom severity [105].

Other important immunomodulatory micronutrients present in breastmilk are vitamins A and D. Vitamin A deficiency, common in low- and middle-income countries, is related to increased mortality and morbidity [106]. The randomized controlled trial (RCT) investigating maternal supplementation with a single megadose vitamin A showed decreased morbidity in exclusively breastfed infants [107]. However, a recent Cochrane review stated that there is no convincing evidence that vitamin A supplementation could reduce morbidity and mortality in infants in the first sixth months of life [108].

#### 3.5.2. Breastfeeding and Systemic Inflammation 

Inflammation is an essential part of an immune response [109]. However, maintaining homeostasis between inflammation and its modulation is crucial. In infancy, both the immune system and the anti-inflammatory mechanisms in the respiratory and gastrointestinal tracts are immature [96]. Breastfeeding may also help infants in maintaining this homoeostasis. A study conducted by Kainonen et al. [110] showed that exclusively breastfeeding promotes an anti-inflammatory cytokine milieu, which is maintained throughout infancy. Breastfeeding exclusivity was also inversely associated with gut inflammation in infants [111]. Moreover, the anti-inflammatory effect of breastfeeding may persist into adolescence [112] and adulthood [113,114], independent of other risk factors; however, not every study found this association [115,116].

The proposed explanation of the anti-inflammatory action of breastfeeding is that breastmilk contains a variety of the aforementioned immunoactive factors and immunomodulators. Lactoferrin, lactoperoxidase, osteopontin, immunoglobins, superoxide dismutase (SOD), platelet-activating factor acetylhydrolase (PAF), alkaline phosphatase, TGF-β, and many of the growth factors also have anti-inflammatory effects [96]. Breastmilk provides a variety of antioxidant compounds that mediate the anti-inflammatory effects in breastmilk. Oxidative stress is an important factor contributing to inflammation. It is worth noticing that infants, especially preterm babies, are prone to oxidative stress due to extensive metabolic activity and higher oxygen in the extrauterine environment; they also have an immature antioxidant defense system [117,118]. It was shown that breastmilk can suppress oxidative stress and diminish oxidative damage in newborns more effectively than infant formula [119]. Breastmilk lactoferrin, lysozyme, glutathione peroxidase (GPx), SOD, catalase, ceruloplasmin, coenzyme Q10, thioredoxin, leptin, and adiponectin are also important breastmilk antioxidants [96]. Moreover, a variety of breastmilk micronutrients and bioactive factors, such as vitamins A, C, and E; iron; copper; zinc; selenium; carotenoids; and flavonoids, have antioxidant properties [96,118,120,121,122]. Several of those nutrients, such as β-carotene, lutein, and α-tocopherol, act both locally and systematically after absorption [120,121]. In vitro and in vivo studies showed that *n*-3 LC PUFAs and vitamins C and E reduced oxidative stress and inflammation due to PM_2.5_ exposure [123,124,125], whereas dietary supplementation with vitamins C and E may mitigate the respiratory and inflammatory effects of O_3_ and PM_10_ in asthmatic children [126,127]. Lutein is a carotenoid that deserves special attention due to the fact that a substantial body of evidence indicates it plays a significant role in the protection against oxidative stress in early postnatal life [128]. Breastmilk antioxidant concentrations and total antioxidant capacity are also the highest in the colostrum and decline with lactation, but is still higher than infant formula [118,121,129,130].

#### 3.5.3. Effects of Breastfeeding on the Respiratory System

A large body of evidence suggests that breastfeeding may protect against adverse respiratory events (e.g., respiratory tract infections [9,89,90], asthma [85,131]) and has a favorable association with lung development and function in later life [132,133,134,135]. However, some studies did not confirm those associations [88,135]. It was reported that breastfed children have larger normalized lung volumes and alveolar size, higher forced vital capacity (FVC), forced expiratory volume (FEV_1_), and mid-expiratory flows (FEF_50_) [132,133,135].

The mechanisms explaining breastfeeding’s action on the respiratory system are not clear; however, several potential mechanisms were proposed. First, breastfeeding may enhance lung development and function throughout the immune-mediated pathway and aforementioned immunoactive factors [134]. Also, lower respiratory infections are associated with reduced lung function and asthma occurrence [13,134,135]. Second, breastmilk cytokines and maternal immunoglobins may reduce or inhibit airway inflammation [132]. This mechanism may be especially plausible as air pollution induces systemic inflammation, and if breastfeeding is able to reduce it, it may protect the bronchial tree against damage [13]. Third, breastfeeding is associated with better anthropometric development, i.e., lower weight gain in infancy, subsequent risk of becoming overweight, and greater attained height [9]. Those factors are important predictors of lung function and asthma risk [134,136,137]. Fourth, breastmilk cytokines and growth factors (e.g., TNF-β1, insulin-like growth factor 1 (IGF-1)) may stimulate lung growth and development [135,138]. Moreover, studies conducted among healthy adults showed that carotenoid status (β-carotene, lutein/zeaxanthin), as well as polyphenol and anthocyanin intake, influence lung efficiency, and those nutrients are present in breastmilk [118,139,140,141]. Fifth, epigenetic mechanisms may be involved in the protective breastfeeding effect as breastfeeding influences DNA methylation [142]. Sixth, the mechanical explanation was proposed in terms of suckling patterns and diaphragmatic movements during breastfeeding, which may contribute to better development of the respiratory muscles and improve the coordination between swallowing and breathing, and therefore, increase lung capacity [13,143,144].

#### 3.5.4. Breastfeeding and Neurodevelopment

Substantial evidence also indicates that breastfeeding contributes to better neurodevelopment and psychomotor and cognitive performance and its effect persists over time [9,145,146]. Thus far, it was shown that breastfed children not only obtained better scores on cognitive tests, but also had better brain structural development and physiological activity [147,148,149]. Breastfeeding may improve neurodevelopment in several ways. One of the proposed mechanisms covers the neuroprotective effect of breastfeeding, which seems to be crucial as air pollution triggers neuroinflammation [20]. Breastmilk contains a variety of factors that reduce oxidative stress and inflammation in the brain, including LC PUFA and antioxidants [12,71,72,150,151]. Several studies investigating the effect of breastfeeding on air pollution’s adverse neurodevelopmental outcomes showed the protective effect of maternal fruit and vegetable consumption due to higher intake of dietary antioxidants [71,72,74]. Fruits and vegetables are dietary sources of various antioxidants, including carotenoids and flavonoids, that are absent in infant formula and present in breastmilk in a diet-dependent manner [118,121]. Dietary intervention among school-aged children showed that flavanols may act as neuroprotective agents from the effects of air pollution exposure [150]. Moreover, breastmilk contains compounds essential for brain development, intracellular communication, and neurotransmission, i.e., LC PUFAs, brain-derived neurotrophic factor (BDNF), glial cell line-derived neurotrophic factor (GDNF), choline, gangliosides and sialic acid, lutein and zeaxanthin, and flavonoids [118,121,146,149,152,153,154,155,156,157]. The influence of other maternal factors, such as socioeconomic and psychosocial factors, are not without significance; however, the effect of breastmilk nutrients and bioactive factors is not diminished [146,158].

Summarizing, it seems that the most plausible and most important mechanisms responsible for the protective effects of breastfeeding against air pollution are the immunomodulatory, antioxidant, and anti-inflammatory properties of breastmilk. Hence, breastmilk nutrients and bioactive factors may significantly contribute to reducing the adverse effects of air pollution. It is important to understand the factors that determine its breastmilk levels and to highlight easily modifiable factors. 

#### 3.5.5. Nutritional Determinants of Breastmilk Composition

Breastmilk composition may be influenced by several factors. It was shown that the levels of the aforementioned immunoactive and antioxidant breastmilk compounds is related to the lactation stage and may be affected by preterm delivery [130,159,160], mode of delivery [160,161], maternal allergy or atopy [160], overweightness and obesity [160,162,163,164], depression [165], physical activity [166,167,168], and maternal tobacco smoking [92,169], as well as place of residence or origin [170,171,172,173], parity [161,171], education, and age [174]. Moreover, the influence of maternal diet on breastmilk composition was widely investigated because it is easily modifiable through dietary interventions. 

Despite the fact that maternal nutrition has a limited influence on breastmilk macronutrients, the fatty acids profile and levels of several micronutrients and bioactive factors with immunomodulatory and antioxidant effects are diet-dependent (i.e., vitamins D, A, C, and B; selenium; carotenoids; and flavonoids) [98,118,121,164,174,175,176]. Previous studies confirmed that safe and cost-effective dietary interventions during pregnancy and lactation using dietary supplements or food products are related to an increase in breastmilk levels of LC PUFAs [177,178,179,180]; vitamins D, A, and B [181,182]; carotenoids [183,184,185]; or flavonoids [186,187,188,189], as well as their antioxidant properties [190,191].

Additionally, dietary interventions may also alter breastmilk cytokines or immunoglobins; however, many results were inconclusive [179,192,193,194,195,196,197,198,199,200,201,202,203,204,205,206]. Several studies did not confirm the influence of fish oil or farmed salmon supplementation on breastmilk TGF-β1, TGF-β2, soluble cluster of differentiation (sCD14), and sIgA levels [179,200,201], but one study found that salmon consumption decreased sIgA levels [179]. Linnamaa et al. [194] showed that blackcurrant seed oil supplementation increased Interferon gamma (INF-γ) and decreased interleukin 4 (IL-4) levels, but had no effect on IL-5, IL-10, IL-12, or TNF levels. More studies investigated the associations between probiotic supplementation and breastmilk immune compounds; however, the results are mixed. It was shown that IL-10 levels increased after the supplementation of *Lactobacillus reuteri* ATCC 55730 [203], *L. rhamnosus* and *Bifidobacterium lactis* Bb12 [205], *L. rhamnosus* GG and LC705, and *B. breve* Bb99 and *Propionibacterium freudenreichii* ssp. *Shermanii* JS [206], whereas another study showed no effect from *L. rhamnosus* HN001 or *B. lactis* HN019 [204]. A decrease in TGF- β2 levels was shown after *L. reuteri* ATCC 55730 supplementation [203], but symbiotic supplementation resulted in its increase [195], and probiotic milk [196], *L. rhamnosus* HN001 [197] or *L. casei* LC5, *B. longum* BG7, and *Bacillus coagulans* SANK70258 [198] had no influence. No study, despite one investigating *L. rhamnosus* HN001 and *B. lactis* HN019 [204], confirmed the influence on TGF-β1 levels [195,196,197,198,203,207]. One study found that probiotic supplementation (*L. rhamnosus* GN) resulted in a decrease in sCD14 concentrations [207], but others found no results [203,204,208]. Studies on the influence of *L. rhamnosus* HN001, GN or *L. casei* LC5, *B. longum* BG7, and *Bacillus coagulans* SANK70258 supplementation on IgA concentrations were also inconclusive [198,204,207], whereas symbiotic supplementation increased IgA levels [195]. The influence of maternal β-carotene or retinol supplementation on sIgA, lactoferrin, lysozyme, and IL-8 was also investigated; however, the authors did not observe any significant associations [192]. On the other hand, the same authors showed that supplementation with vitamin-E-rich sunflower oil but not rich in provitamin A red palm oil decreased breastmilk IL-8 and TGF-β levels [199]. In contrast, it was shown that retinyl palmitate supplementation during pregnancy may increase colostrum sIgA levels [193]. A study from Japan investigating *Chlorella* supplementation during pregnancy showed an increase in breastmilk IgA levels and a reduction in dioxin transfer into milk [202]. Differences in the assessed outcomes may be the result of different study protocols. Further studies investigating the influence of maternal supplementation on breastmilk immunological properties are necessary. 

Moreover, it is noteworthy that previous studies conducted among adults showed that dietary supplementation with LC PUFAs or antioxidants may diminish the adverse health effects of air pollution [7,209]. Hence, these kinds of interventions could also be beneficial for mothers exposed to air pollution. 

### 3.6. The Influence of Air Pollution on Breastmilk Composition

Previous studies that did not find a modifying effect of breastfeeding on the health effects induced by air pollutants hypothesized that it may be related to chemical contamination or the transfer of inflammatory molecules or signals of air pollution exposure via breastmilk of mothers exposed to air pollution [74,83]. Only a few studies described the associations between air pollution exposure and breastmilk chemical contamination by PAHs and heavy metals (Table 4). 

However, none of those studies quantitatively assessed maternal exposure and compared breastmilk chemical levels with pollution levels (e.g., from urban and rural areas). The study groups were small, maternal detailed exposure was not assessed, and breastmilk was usually collected only at one time-point. However, breastmilk chemical assessment may be an effective and non-invasive tool to estimate maternal and infant exposure to environmental pollutants [210]. According to the existing literature, breastmilk PAHs levels seem to be associated with the air pollution level in the maternal place of residence, which has not been confirmed in the case of heavy metals. The source of those chemicals is not only air pollution but also water and soil pollution [211,212]. Moreover, factors such as maternal smoking [211,213,214], diet [211,214], maternal obesity [215], age [214,216], education [217], or parity [214] may affect their levels in breastmilk [218].

Poniedziałek et al. [122] investigated the association between pollution in the maternal residential area, breastmilk chemical contamination, and bioactive factors. The authors found that breastmilk total polyphenols were adversely associated with breastmilk malonodialdehyde and aluminum, but no other heavy metals, as well as positively with total antioxidant capacity [122]. Studies investigating the influence of maternal tobacco smoking on breastmilk composition provided more observations. Maternal tobacco smoking impairs breastmilk taste and composition, including levels of breastmilk antioxidants, LC PUFAs, cytokines, and immunoglobin profile, as well as heavy metals [223]. Hence, exposure to tobacco smoke is associated with similar biological mechanisms involving oxidative stress and inflammation. We hypothesized that similar changes may occur in breastmilk composition affected by air pollution. Thus far, only one animal study analyzed the influence of exposure to diesel exhaust during pregnancy on mammary gland development and breastmilk composition [224]. The authors found that maternal exposure did not alter mammary gland histology and leptin expression, but affected the expression of the *stearoyl-CoA desaturase* (*SCD*) gene involved in lipid metabolism, and therefore, an alteration of the milk lipid and protein profile was found [224]. Moreover, studies on maternal tobacco smoking showed that mothers who smoked produced less milk and breastfed for a shorter time, probably due to changes in hormone levels; affected suckling in infants; and had a higher risk of lactation problems (e.g., mastitis) [223]. Further studies investigating those issues are necessary. 

Breastmilk contamination by environmental pollutants, including those from the air, is related to infant exposure not only through the air, but also through breastmilk. Levels of breastmilk contaminants vary among women with different exposures and life histories. Usually, those levels are the highest in women from developing countries living in a harsh environment and exposed to higher doses of pollutants [210]. This decreased the safety of breastmilk, even though it provides nourishment. Also, environmental contaminants may raise maternal fears and discourage women from breastfeeding due to concerns about their infant’s health [225]. However, it should be highlighted that baby foods and infant formulas may also be a source of chemical contaminants, both in themsevles and through the water used to prepare them [210,226,227,228]. In addition, infant formulas contain only a limited number of bioactive factors with constant values that cannot be compared with breastmilk’s bioactive values [229]. It should be highlighted that a variety of studies investigating breastmilk contamination by environmental pollutants reported that the breastfeeding benefits outweigh the potential health risk to the infant [218]. Thus far, most studies investigating the possible modifying effect of breastfeeding on adverse health outcomes of air pollution exposure confirmed its protective action. Moreover, breastmilk is a completely natural, renewable, and free food that does not need packaging and does not generate unnecessary waste. This makes breastfeeding widely available and environmentally safe. Therefore, breastfeeding’s contribution to sustainability and food security should be considered in environmental goals and policies [230,231].

## 4. Conclusions

The current literature suggests that breastfeeding may diminish the adverse effects of air pollution in immune, respiratory, nervous, and cardiovascular system, as well as under-five mortality. It was proposed that this protective influence may be a result of the antioxidant, anti-inflammatory, and immunomodulatory properties of breastmilk that diminish the damage induced by air pollutants, as well as the beneficial influence on immune, respiratory, and nervous systems. Nutrients and bioactive agents, such as LC PUFAs, antioxidant vitamins, carotenoids, flavonoids, growth factors, and immune factors (e.g., immunoglobulins, cytokines) may be responsible for the protective influence of breastmilk. Nevertheless, maternal exposure to air pollution may increase the levels of chemical contaminants in breastmilk. However, it should be highlighted that despite the risks associated with the chemical contamination of breastmilk, mothers should be encouraged to breastfeed their infants. Thus far, it was shown that breastfeeding benefits outweigh its potential health risks. Moreover, breastfeeding has a limited ecological footprint and its contribution to sustainability and food security should be considered in environmental goals and policies. Further prospective cohort studies investigating the associations between breastfeeding and health outcomes under air pollution exposure conditions are necessary. 

## Figures and Tables

**Table 1:** Health effects of air pollution exposure in the first 1000 days of life.

Health Effects		Air Pollutants		Reference
		Exposure	Type	
Birth outcomes	Orofacial clefts	Prenatal	O_3_	[56]
	Premature birth	Prenatal	PM_2.5_, PM_10_, NO_2_, CO, PAHs	[19]
	Low birth weight	Prenatal	PM_2.5_, PM_10_, NO_2_, SO_2_, CO, PAHs	[19]
	IUGR	Prenatal	PM_2.5_, PM_10_, NO_2_, CO, PAHs, B(a)P	[19,23]
Short-term effects	Lower respiratory illness	Prenatal, postnatal	PM_2.5_, PM_10_, NO_2_, NOx, CO, PAHs, benzene, household air pollution	[17,19,57,58,59]
	Pneumonia	Prenatal, postnatal	PM_10_, Zn in PM_10_, NO_2_,	[19,60,61]
	Otitis media	Postnatal	PM_2.5_, NOx, CO	[59]
Long-term effects	Asthma	Prenatal, postnatal	PM_2.5_, PM_10_, NO_2_, SO_2_, PAHs	[19,42,62,63,64]
	Allergic rhinitis	Prenatal, postnatal	PM_2.5_, PM_10_, NO_2_	[46,63,64]
	Eczema	Prenatal	NO_2_	[19]
	Overweight and obesity	Prenatal, postnatal	PM_2.5_, PM_10_, UFP, NO_2_, BC	[35,65]
	Type 1 diabetes	Postnatal	PM_2.5_, PM_10_, O_3_, SO_4_	[30,66]
	Affected pubertal development	Prenatal, postnatal	PM_10_, NO_2_, SO_2_	[34]
	Blood pressure and hypertension	Postnatal	PM_10_, O_3_	[45,48,67]
	Reduced arterial distensibility	Postnatal	PM_2.5_, PM_10_, NO_2_, NO_x_	[54]
	Irritable bowel syndrome	Postnatal	CO, NO_2_, NMHC, CH_4_	[24]
	Reduced pulmonary function	Prenatal, postnatal	PM_10_, NO_2_, benzene	[13,19,68,69]
	Brain structural alterations	Prenatal	PM_2.5_	[52]
	Poorer cognitive development	Prenatal, postnatal	PM_2.5_, PM_10_, NO_2_, SO_2_, BC, PAHs, benzene, NHMC, household air pollution	[12,51,70,71,72,73,74]
	Adolescent psychotic experiences	Postnatal	NO_2_	[75]
	ADHD	Postnatal	PM_10_	[22]
	Autism spectrum disorder	Prenatal	PM_2.5_, PM_10_	[76]
	Childhood cancers	Postnatal	PM_10_, NO_2_, benzene, B(a)P	[77]
	SIDS	Postnatal	NO_2_, SO_2_	[78]
	Childhood mortality	Prenatal, postnatal	Household air pollution	[79,80,81]

ADHD—attention deficit and hyperactivity disorder, B(a)P—benzo(a)pyrene, BC—black carbon, IUGR—intrauterine growth restriction, NHMC—nonmethane hydrocarbon, PAHs—polycyclic aromatic hydrocarbons, SIDS—sudden infant death syndrome, UFP—ultrafine particles.

**Table 2:** Studies investigating the influence of breastfeeding on health effects induced by indoor air pollution exposure in the first 1000 days of life.

Design	Population	Sample Size	Air Pollutants		Breastfeeding Definition	Main Outcome Measures	Age at Measurement	Interpretation	Reference
			Type	Exposure					
Cohort study (AMICS)	United Kingdom and Spain	*n* = 1611	• NO_2_	2 weeks at age 3 months	Duration of any BF	LRI	1st year of life	BF had no modifying effect	Sunyer et al. [57]
Cohort study (Teplice Program)	Czech Republic	*n* = 452	• Air pollution from coal fuels and smoking	Prenatal period	Ever or never BF	LRI	3 years of life	Never BF children had a higher risk of LRI compared to ever BF children	Baker et al. [58]
Case control cross-sectional study	China	*n* = 360	• PM_1_ • PM_2.5_ • PM_10_	2 months previous	Ever or never BF	Serum miR-155 concentrations (asthma risk)	Average 10 years	Ever BF showed protective function for childhood asthma.	Liu et al. [38]
Cohort study (INMA)	Spain	*n* = 1887	• Air pollution from gas cooking	Prenatal period	Any BF <6 or ≥6 months	Mental development	11–22 months of life	Inverse associations between indoor air pollutants and mental development were stronger in children BF for a shorter time	Vrijheid et al. [71]
Cross-sectional study (NDHS)	Nigeria	*n* = 38,522	• Air pollution from solid fuels	Postnatal	Currently BF	Under-five mortality	0–5 years	Current BF decreased the risk of neonatal and postnatal mortality.	Ezeh et al. [79]
Cross-sectional study (DHS)	23 sub-Saharan countries	*n* = 783,691	• Air pollution from cooking fuel	Postnatal	Currently BF	Under-five mortality	0–5 years	Current BF increased the risk of death compared to children who had stopped BF	Owili et al. [80]
Cross-sectional study (PDHS)	Pakistan	*n* = 11,507	• Air pollution from cooking fuel	Postnatal	Ever or never BF	Under-five mortality	0–5 years	Ever BF children had lower risk of mortality	Naz et al. [81]

AMICS—Asthma Multicentre Infant Cohort Study; BF—breastfeeding; DHS—Demographic and Health Survey; INMA—Infancia y Medio Ambiente, the Spanish for Childhood and Environment study; LRI—lower respiratory tract infections; NDHS—Nigeria Demographic and Health Survey; PDHS—Pakistan Demographic and Health Survey. Characteristics of the examined studies are presented in Table S1.

**Table 3:** Studies investigating the influence of breastfeeding on health effects induced by outdoor air pollution exposure in the first 1000 days of life.

Design	Population	Sample Size	Air Pollutants		Breastfeeding Definition	Main Outcome Measures	Age at Measurement	Interpretation	Reference
			Type	Exposure					
Cross-sectional study (SNECCS)	China	*n* = 31,049	• PM_10_ • SO_2_ • NO_2_ • O_3_	Previous 3 years	Mainly BF for at least 3 months	Respiratory conditions	2–14 years	BF was associated with smaller associations between air pollution and respiratory conditions (cough, phlegm, current wheeze, and asthma), especially in younger children	Dong et al. [11]
Cohort study (BILD)	Switzerland	*n* = 436	• PM_10_	Lifetime exposure	Duration of any BF	Respiratory symptoms	First 27 weeks of life	PM_10_ had a stronger nonsignificant effect on the occurrence of respiratory symptoms in BF infants	Gorlanova et al. [83]
Cross-sectional study (CCHH)	China	*n* = 30,759	• PM_2.5_ (at kindergarten)	Previous year	Any BF ≤6 or >6 months	Asthmatic and allergic symptoms	4.6 years	BF shorter than 6 months was associated with higher odds of doctor-diagnosed asthma and allergic rhinitis	Chen et al. [63]
Cross-sectional study	China	*n* = 39,782	• PM_10_ • NO_2_ • SO_2_	Lifetime exposure	Ever or never BF	Asthma, rhinitis, and respiratory symptoms	3–6 years	BF was negatively associated with doctor-diagnosed asthma, rhinitis	Norbäck et al. [64]
Cross-sectional study (SNECCS)	China	*n* = 6740	• PM_1_ • PM_2.5_ • PM_10_ • NO_2_	Previous 4 years	Mainly BF for at least 3 months	Lung function	7–14 years	BF was associated with a lower risk of lung function impairment induced by air pollutants, especially in the younger group (<12 years)	Zhang et al. [13]
Cross-sectional study (SNECCS)	China	*n* = 9354	• PM_10_ • SO_2_ • NO_2_ • O_3_• CO	Previous 4 years	Mainly BF for at least 3 months	Blood pressure	5–17 years	Never BF children exposed to PM_10_, O_3_, CO, and NO_2_ had higher odds of hypertension compared to BF children. There were no significant associations between BF, air pollution, and blood pressure	Dong et al. [84]
Cohort study (INMA)	Spain	*n* = 1889	• NO_2_ • benzene	Prenatal period	Any BF never; <6 or ≥6 months	Mental development	11–23 months of life	Inverse associations between air pollutants and mental development were stronger in never BF infants, but effect estimates and interactions were not significant	Guxens et al. [72]
Cohort study	Poland	*n* = 170	PAHs	Prenatal	Exclusive BF (EBF; WHO definition)	Mental development	7 years	EBF for at least 6 months decreased the risk of depressed verbal IQ	Jedrychowski et al. [73]
Cohort study (INMA)	Spain	*n* = 438	• PM_2.5_ • NO_2_ • benzene	Prenatal	EBF ≤4 or >4 months	Mental development	2nd year of life	EBF modified the adverse effect between PM_2.5_ and NO_2_ exposure and mental score; any BF had no effect	Lertxundi et al. [74]
Cohort study (INMA)	Spain	*n* = 1119	• PM_2.5_ • NO_2_	Prenatal period	Duration of predominant BF	Neuropsychological development	4–6 years	BF had no modifying effect on the adverse association between exposure to air pollution and domains related to memory, verbal, and general cognition	Lertxundi et al. [12]

BF—breastfeeding; BILD—Bern–Basel Infant Lung Development; CCHH—China, Children, Homes, and Health study; EBF—exclusive breastfeeding; INMA—Infancia y Medio Ambiente, the Spanish for Childhood and Environment study; IQ—intelligence quotient; PAHs—polycyclic aromatic hydrocarbons; SNECCS—Seven Northeastern Cities Chinese Children’s Study. Characteristics of the examined studies are presented in Table S2.

**Table 4:** Studies investigating the influence of maternal air pollution exposure on breastmilk chemical contamination.

Study Group and Time	Exposure	Assessed Compound	Breastmilk Sample	Breastmilk Levels	Reference
*n* = 32 Italy (Tuscany) 2004–2005	• Residential area (urban area highly polluted)	PAHs	Mature sample (78%) 15–25 mL	• ↑ in urban vs. rural area	Zanieri et al. [213]
*n* = 162 Czech Republic (North Moravian; Bohemia) 2013–2014	• Two residential areas with different pollution • Winter and summer period	PAHs	15–30 mL	• ↑ PHE, FLN, B(a)P in higher polluted vs. lower polluted area	Pulkrabova et al. [219]
*n* = 126 China (Lanzhou) 2016	• Residential area	PAHs	Within 43 days postpartum 20–40 mL	• ↑ in industrial vs. residential area	Wang et al. [211]
*n* = 128 Ghana (Accra) 2014–2016	• Residential area (area near e-waste recycling site)	PAHs	Mature milk	• ↑ in e-waste vs. residential area	Asamoah et al. [212]
*n* = 89 Malaysia (Kuala Lumpur; Kuala Langat) 2014–2016	• Residential area	Pb	Mature milk 10–20 mL	• ↑ in urban vs. rural area	Huat et al. [220]
*n* = 54 Italy lack of information	• Residential area	Pb	Mature milk	• ↑ in urban vs. rural area	Guidi et al. [221]
*n* = 29 Croatia (Northern Adriatic Area) 1995–1996	• Residential area	Cd, Pb	2–12 days postpartum ≈80 mL	• ↑ Pb in urban vs. rural area	Frković et al. [222]
*n* = 95 Greece 2000–2002	• Residential area	Zn, Fe, Cu, Mn, Cd, Pb	3 and 14 days postpartum	• ↑ Pb in urban vs. rural area	Leotsinidis et al. [217]
*n* = 120 India (Accra) 2014–2016	• Residential area (area near integrated steel plant)	As, Pb, Mn, Hg, Cd	Colostrum	• ↑ in integrated steel plant vs. residential area	Sharma et Pervez [216]
*n* = 100 Spain (Madrid) 2003–2004	• Exposure to motor vehicle traffic	Hg, Pb, Cd	Mature milk 20 mL	• Pb was associated with exposure	García-Esquinas et al. [214]
*n* = 75 Poland (Poznań) 2016	• Residential area	Al, As, Cd, Ni, Pb	Colostrum 15–20 mL	• No associations with place of residence	Poniedziałek et al. [122]

Al—aluminum, As—arsenic, B(a)P—benzo(a)pyrene, Cd—cadmium, Cu—copper, Fe—iron, FLN—fluorene, Hg—mercury, Mn—manganese, Ni—nickel, PAHs—polycyclic aromatic hydrocarbons, Pb—lead, PHE—phenanthrenel, Zn—zinc, ↑—higher concentration.

## Supplementary Materials

The following are available online at https://www.mdpi.com/1660-4601/16/21/4181/s1. Table S1: Characteristics of studies investigating the influence of breastfeeding on health effects induced by indoor air pollution exposure in the first 1000 days of life. Table S2: Characteristics of studies investigating the influence of breastfeeding on health effects induced by outdoor air pollution exposure in the first 1000 days of life.

## Abbreviations

ADHD	attention deficit and hyperactivity disorder
AMICS	Asthma Multicentre Infant Cohort Study
AQG	Air Quality Guide
Al	aluminium
As	arsenic
B(a)P	benzo(a)pyrene
BC	black carbon
BDNF	brain-derived neurotrophic factor
BF	breastfeeding
BILD	Bern–Basel Infant Lung Development
CCHH	China, Children, Homes, and Health study
Cd	cadmium
CD14	cluster of differentiation 14
CO	carbon monoxide
Cu	copper
DHS	Demographic and Health Survey
EBF	exclusive breastfeeding
ET-1	endothelin-1
Fe	iron
FEF_50_	mid-expiratory flows
FEV_1_	forced expiratory volume
FLN	fluorene
FVC	forced vital capacity
GDNF	glial cell line-derived neurotrophic factor
GDP	gross domestic product
GPx	glutathione peroxidase
Hg	mercury
IgA, IgM, IgG	immunoglobins A, M, G
IGF-1	insulin-like growth factor 1
INMA	Infancia y Medio Ambiente, the Spanish for Childhood and Environment study
IUGR	intrauterine growth restriction
IQ	intelligence quotient
LC PUFA	long chain polyunsaturated fatty acids
LRI	lower respiratory tract infections
Mn	manganese
NDHS	Nigeria Demographic and Health Survey
Ni	nickel
NMHC	nonmethane hydrocarbon
NO_x_	nitrogen oxides
O_3_	ozone
OECD	Organisation for Economic Co-operation and Development
PAF	platelet-activating factor acetylhydrolase
PAHs	polycyclic aromatic hydrocarbons
Pb	lead
PDHS	Pakistan Demographic and Health Survey
PM	particulate matter
PRISMA	Preferred Reporting Items for Systematic Reviews and Meta-Analyses
PROBIT	Promotion of Breastfeeding Intervention Trial
RCT	randomized controlled trial
sCD14	soluble cluster of differentiation 14
SCD	stearoyl-CoA Desaturase
SIDS	sudden infant death syndrome
sIgA	secretory immunoglobin A
SNECCS	Seven Northeastern Cities Chinese Children’s Study
SO_2_	sulfur dioxide
SOD	superoxide dismutase
TGF-β	transforming growth factor β
TNF-β	tumor necrosis factor β
UFP	ultrafine particles
UVB	ultraviolet B
WHO	World Health Organization
Zn	zinc
