# Supplementary material for: Protective Effect of Breastfeeding on the Adverse Health Effects Induced by Air Pollution: Current Evidence and Possible Mechanisms
Source: Int J Environ Res Public Health. 2019 Oct 29;16(21):4181. doi: 10.3390/ijerph16214181 (PMC6862650; doi:10.3390/ijerph16214181)
Supplement: Supplementary file 1 [file ijerph-16-04181-s001.pdf]

International Journal of
Environmental Research and Public Health

MDPI

Protective Effect of Breastfeeding on the Adverse Health Effects Induced by Air Pollution: Current Evidence and Possible Mechanisms

Monika A. Zielinska [1]* and Jadwiga Hamulka [1]

Table S1. Characteristics of studies investigating the influence of breastfeeding on health effects induced by indoor air pollution exposure in the first 1,000 days of life.

Study	Air pollutants			Breastfeeding		Main Outcome			Data analysis
	Type	Exposure	Measurement	Definition	Data collecting	Type	Age	Measurement	
CS (AMICS) UK and Spain *n* = 1611 [57]	• NO_2	2 weeks at age 3 months	Passive diffusion tubes used for measurements in living room, colorimetric reaction	Duration of any BF	Questionnaire (duration of any BF)	Doctor-diagnosed LRI	1st year of life	Questionnaire and examination of clinical records	Logistic regression analysis used to assess the association between AP exposure and LRI infections adjusted on BF duration
CS (Teplice Program) Czech Republic *n* = 452 [58]	• AP from coal fuels and smoking	Prenatal period	Questionnaire (indoor heating and cooking fuel sources)	Ever or never BF	Questionnaire (duration of exclusive and any BF)	Doctor-diagnosed LRI	3 years of life	Examination of clinical records	Generalized estimating equations used to investigate the associations between LRI and coal heating or maternal smoking separately in ever or never BF children
CC CSS Study China *n* = 360 [38]	• PM_1 • $PM_{2.5}$ • PM_{10}	2 months previous	Handheld particle counter used for measurements in living room and bedroom	Ever or never BF	questionnaire	Serum miR-155 concentrations	Average 10 years	Analysis by real-time PCR in asthmatic and healthy children	Logistic regression analysis used to assess the associations between AP and the risk of asthma and serum miR-155 level. BF was one of the predictors.

Table S1. Cont.

Study	Air pollutants			Breastfeeding		Main Outcome			Data analysis
	**Type**	**Exposure**	**Measurement**	**Definition**	**Data collecting**	**Type**	**Age**	**Measurement**	
CS (INMA) Spain *n* = 1887 [71]	• AP from gas cooking	Prenatal period	Questionnaire (cooking fuel sources)	Any BF <6 or ≥ 6 months	questionnaire	Mental development	11–22 months of life	Bayley Scales of Infant Development	Linear regression used to examine the association between gas cooking and mental development score. BF was one of the predictors.
CSS (NDHS) Nigeria *n* = 38,522 [79]	• AP from solid fuels	Postnatal	Face-to-face interview (cooking fuel sources and kitchen location)	Currently BF	questionnaire	Under-five mortality	0–59 months	Self-reported by mother	Cox proportional hazards regression analysis used to examine the effect of several predictors (incl. BF) on mortality
CSS (DHS) 23 Sub-Saharan countries *n* = 783,691 [80]	• AP from cooking fuel	Postnatal	Face-to-face interview (cooking fuel sources and kitchen location)	Currently BF	questionnaire	Under-five mortality	0–59 months	Self-reported by mother	Cox proportional hazards regression analysis used to examine the effect of several predictors (incl. BF) on mortality
CSS (PDHS) Pakistan *n* = 11,507 [81]	• AP from cooking fuel	Postnatal	Face-to-face interview (cooking fuel sources and kitchen location)	Ever or never BF	questionnaire	Under-five mortality	0–59 months	Slf-reported by mother	Logistic regression analysis used to assess the effect of several predictors (incl. BF) on the risk of mortality

AMICS – Asthma Multicentre Infant Cohort Study; AP – air pollution; BF – breastfeeding; CC – case-control; CS – cohort study; CSS – cross-sectional study; DHS – Demographic and Health Survey; INMA – INfancia y Medio Ambiente, the Spanish for Childhood and Environment study; LRI –lower respiratory tract infections; NDHS – Nigeria Demographic and Health Survey; PDHS – Pakistan Demographic and Health Survey; UK – United Kingdom.

Table S2. Characteristics of studies investigating the influence of breastfeeding on health effects induced by outdoor air pollution exposure in the first 1,000 days of life.

Design	Air pollutants			Breastfeeding		Main Outcome			Data analysis
	**Type**	**Exposure**	**Measurement**	**Definition**	**Data collecting**	**Type**	**Age**	**Measurement**	
CSS (SNECCS) China *n* = 31,049 [11]	• PM_{10} • SO_2 • NO_2 • O_3	Previous 3 years	Calculated based on data from monitoring stations	Mainly BF for at least 3 months	Questionnaire	Respiratory conditions	2–14 years (previous 12 months)	Questionnaire	Logistic regression analysis used to examine the effect of AP exposure separately in ≥ 3 mo and ≤ 3 mo BF children
CS (BILD) Switzerland *n* = 436 [83]	• PM_{10}	Lifetime exposure	Calculated based on data from monitoring stations	Duration of any BF	Telephone interview	Respiratory symptoms	First 27 weeks of life	Telephone interview using a standardized symptom score	Generalized additive mixed model used to examine the association between AP exposure, several predictors (incl. BF) and respiratory symptoms also separately in BF and NBF children
CSS (CCHH) China *n* = 30,759 [63]	• $PM_{2.5}$ (at kindergarten)	Previous 1 years	Calculated based on GDP data	Any BF ≤ 6 or > 6 months	Questionnaire	Asthmatic and allergic symptoms	4.6 years (previous 12 months)	Questionnaire	Logistic regression analysis used to examine the association between AP exposure and symptoms. BF was one of predictors.
CSS China *n* = 39,782 [64]	• PM_{10} • NO_2 • SO_2	Lifetime exposure	Calculated based on annual data for each city	Ever or never BF	Questionnaire	Asthma, rhinitis and respiratory symptoms	3–6 years	Questionnaire	Logistic regression analysis used to examine the association between AP exposure and symptoms. BF was one of the predictors.

Table S2. Cont.

Design	Air pollutants			Breastfeeding		Main Outcome			Data analysis
	Type	Exposure	Measurement	Definition	Data collecting	Type	Age	Measurement	
CSS (SNECCS) China *n* = 6740 [13]	• PM_1 • $PM_{2.5}$ • PM_{10} • NO_2	Previous 4 years	Calculated based on data from monitoring stations	Mainly BF for at least 3 months	Questionnaire	Lung function	7–14 years	Spirometry	Logistic regression analysis used to examine the association between AP exposure and lung function separately in BF and NBF children
CSS (SNECCS) China *n* = 9354 [84]	• PM_{10} • SO_2 • NO_2 • O_3 • CO	Previous 4 years	Calculated based on data from monitoring stations	Mainly BF for at least 3 months	Questionnaire	Blood pressure	5–17 years	Measurement	Logistic regression analysis used to examine the association between AP exposure and hypertension and blood pressure separately in BF and NBF children
Cohort study (INMA) Spain *n* = 1889 [72]	• NO_2 • benzene	Prenatal period	Calculated based on data from monitoring stations	Any BF never; <6 or ≥ 6 months	Face-to-face interview	Mental development	11–23 months of life	Bayley Scales of Infant Development	Linear regression used to examine the association between AP exposure and mental development score. BF was one of the predictors.
CS Poland *n* = 170 Jedrychowski et al. [73]	PAHs	Prenatal	Exposure measured by cord blood PAH-DNA adducts	Exclusive BF (EBF; WHO definition)	Face-to-face interview	Mental development	7 years	Wechsler Intelligence Scale for Children-Revised	Binary regression used to examine the association between PAH-DNA adducts and verbal IQ score. BF was one of the predictors.

Table S2. Cont.

Design	Air pollutants			Breastfeeding		Main Outcome			Data analysis
	**Type**	**Exposure**	**Measurement**	**Definition**	**Data collecting**	**Type**	**Age**	**Measurement**	
CS (INMA) Spain *n* = 438 [74]	• $PM_{2.5}$ • NO_2 • benzene	Prenatal	Calculated based on data from monitoring stations	EBF ≤ 4 or > 4 months	Face-to-face interview	Mental development	2nd year of life	Bayley Scales of Infant Development	Linear regression used to examine the association between AP exposure and mental development score. BF was one of the predictors.
CS (INMA) Spain *n* = 1119 [12]	• $PM_{2.5}$ • NO_2	Prenatal period	Calculated based on data from monitoring	Predominant BF ≤ 4 or > 4 months	Face-to-face interview	Neuropsychological development	4–6 years	McCarthy scales	Linear regression used to examine the association between AP exposure and mental development score. The analysis was stratified by BF duration

AP – air pollution; BF – breastfeeding; BILD – Bern-Basel Infant Lung Development; CCHH – China, Children, Homes and Health study; EBF – exclusive breastfeeding; GBD – global burden disease; INMA – INfancia y Medio Ambiente, the Spanish for Childhood and Environment study; IQ – intelligence quotient; NBF – never breastfed; PAHs – polycyclic aromatic hydrocarbons; SNECCS – Seven Northeastern Cities Chinese Children's Study.
